# Risk Factors for Acute Endophthalmitis following Cataract Surgery: A Systematic Review and Meta-Analysis

**DOI:** 10.1371/journal.pone.0071731

**Published:** 2013-08-26

**Authors:** He Cao, Lu Zhang, Liping Li, SingKai Lo

**Affiliations:** 1 Injury Prevention Research Center, Medical College of Shantou University Shantou, Guangdong Province, People's Republic of China; 2 Department of Ophthalmology , No.4 Hospital of Xi'an City, Xi'an, Shanxi Province, People's Republic of China; 3 Faculty of Liberal Arts and Social Sciences, The Hong Kong Institute of Education, Hong Kong, People's Republic of China; 4 Department of Ophthalmology, C-MER(Shenzhen)Dennis Lam Eye Hospital, Shenzhen, Guangdong Province, People's Republic of China; Massachusetts Eye & Ear Infirmary, Harvard Medical School, United States of America

## Abstract

**Background:**

Acute endophthalmitis is one of the most serious complications of cataract surgery and often results in severe visual impairment. Several risk factors for acute postoperative endophthalmitis (POE) following cataract surgery have been reported but the level of evidence and strength of association is varied. The purpose of this study was to critically appraise published reports on and to summarize clinical risk factors associated with acute POE which could be easily assessed by ophthalmologists for the introduction and implementation of preventive measure.

**Methods:**

A systematic review and meta-analysis of observational studies was performed. Six databases were searched with no limits on the year or language of publication. Study-specific odds ratios (Ors) or relative risk (RR) of each risk factor were pooled using a random effect model.

**Results:**

A total of 6 686 169 participants with 8 963 endophthalmitis in 42 studies were analyzed. Of the nine risk factors identified in our systematic review and meta-analysis, extra- or intracapsular cataract extraction, a clear corneal incision, without intracameral cefazolin (1 mg in 0.1 ml solution), without intracameral cefuroxime (1 mg in 0.1 ml solution), post capsular rupture, silicone intraocular lenses and intraoperative complications were found strongly associated with acute endophthalmitis. Other significant factors with a lower strength of association (risk estimates generally 1.5 or less) were male gender and old age (85 years and older).

**Conclusions:**

Our study provides summary data on the risk factors for acute POE. Identifying patients at high risk of this sight-threatening eye disease is important from both the public health and clinical perspectives as this would facilitate detection of disease before the onset of irreversible visual loss enabling earlier intervention.

## Introduction

The World Health Organization's Prevention of Blindness and Visual Impairment makes the global estimate that the number of people of all ages visually impaired is estimated to be 285 million, of whom 39 million are blind in 2010. The major causes of visual impairment are uncorrected refractive errors (43%) and cataract (33%) ; cataracts remain the leading cause of blindness (51%) [1]. Cataract surgery is becoming more prevalent in the elderly as the life expectancy of the population increases. There has been a dramatic shift in surgical practice during the last 30 years with small-incision phacoemulsification being the predominant method of intervention used since 1990. Although cataract surgery is highly effective and relatively safe, owing to the enormous numbers, even uncommon surgical complications could potentially harm many patients. Endophthalmitis is one of the most serious complications of cataract surgery and often results in severe visual impairment [2]. Nationwide surveys and large case series of postcataract endophthalmitis (POE) in different countries estimated that the incidence for endophthalmitis ranged from 0.012% to 1.3% since 2000, in part because of differences in study design, time, and region [3, 17 ∼ 32]. Earlier literatures stratified the results over time and noted decreasing endophthalmitis rates, from 0.327% in the 1970s to 0.158% in the 1980s and 0.087% in the 1990s [4], [43], [57].

The optimal means to prevent POE remains controversial because conducting the large studies required to investigate an uncommon problem is difficult. While preoperative preparation with 5% povidone-iodine solution dropped into the conjunctival sac is the best established method of chemoprophylaxis based on the current clinical evidence, the benefit of other forms of perioperative factors remains uncertain [5], [6], [8], [24], [28], [36]. Several extensive reviews have been written regarding this topic despite the variable evidence and strength of association [6∼11]. A recent meta-analysis found a spike in endophthalmitis rates up to 0.265% during 2000–2003, which might be attributable to the popularization of sutureless clear corneal incisions [4]. Two other studies reported reduced rates of postoperative endophthalmitis among surgeries in the mid- 2000s compared to those performed in the late 1990s, suggesting sutureless incisions may not be the culprit [22], [67]. Herein, we seek to review the most up-to-date evidence and provide our opinion with regard to methods of endophthalmitis prophylaxis for cataract surgery. This meta-analysis identified the patient-related and surgery-related factors that affect the risk for acute POE following cataract.

## Materials and Methods

### Search Strategy

We conducted a systematic review of six databases, including PubMed (1950 to February 1, 2013), EMBASE (1966 to February 1, 2013) , Web of Science (1900 to February 1, 2013), Cochrane library (including the Cochrane Central Register of Controlled Trials, 1800 to February 1, 2013), abstracts from the Association for Research in Vision and Ophthalmology (January 1962 to February 1, 2013), and the National Institutes of Health Clinical Trial Databases [10] (up to February 1, 2013).

These databases were searched systematically using the terms [(endophthalmitis) and (cataract surgery or cataract extraction or cataract or intracapsular cataract extraction or extracapsular cataract extraction or phacoemulsification) and (risk factors or association)]. The search strategy used both keywords and Medical Subject Headings (MeSH) terms. There were no limits placed on the year of publication. All potentially relevant non-English publications were to be translated into English for further assessment. References identified from bibliographies of pertinent articles or books also were retrieved. We followed the criteria used in the Endophthalmitis Vitrectomy Study (EVS) [29], not excluding any presumed acute endophthalmitis after cataract surgery (i.e., including suspected and/or confirmed cases by positive culture), because it would allow for the inclusion of more studies in order to generate more power for analyzing this relatively rare complication.

Retrieved studies were imported into Refworks (version 1.0; Refworks, Bethesda, MD). Duplicate articles appeared twice or more, whether in the same or different databases were deleted. Data extraction and evaluation of study quality were performed independently by two reviewers; any disagreements were resolved by discussion with the senior investigators. The bibliographies of the full text articles that were reviewed were searched for relevant references. Full-text articles were then obtained based on the initial screening of abstracts and the data extraction form was completed. The full texts of the remaining studies were then read to determine whether they met our inclusion criteria. In addition, the reference lists from all identified studies were examined. Potential 45 risk factors for endophthalmitis following cataract surgery were identified in the initial review (Table 1).

**Table 1:** Potential 45 risk factors for endophthalmitis following cataract surgery were identified in the initial review.

**Preoperative risk factors ( n = 13 )**
• Male gender*
• Older individuals ( ≥85 years ) *
• Black race
• Native Americans
• Diabetes mellitus
• Recent tamsulosin exposure ( <14 days before surgery)
• Same day surgery
• Inpatient surgery
• Outpatient surgery
• Dedicated ophthalmic theatre
• Private hospital
• Preoperative topical antibiotics
• Residence inside of city
**Intra-operative risk factors ( n = 26 )**
• Surgeons with low annual volume ( 1∼50 surgeries )
• Surgeons with less experience ( 1∼10years )
• Consultant grade surgeon
• Surgeries performed in 2003
• Face masks not worn in theatre
• Skin disinfection type ( 5% povidone-iodine )
• Conjunctival disinfection type ( without povidone-iodine )
• Topical anesthesia
• Region of cataract surgery: region 6 and 9
• Surgery longer than 45 minutes
• Without intracameral cefazolin*
• Without intracameral cefuroxime*
• Without fourth-generation fluoroquinolones
• Without intracameral vancomycin
• Without subconjunctival antibiotics at the end of surgery*
• Posterior capsular rupture*
• Intraoperative complications*
• A clear corneal incision*
• Silicon based IOL*
• Polymethyl methacrylate based IOL
• Foldable IOL
• Communication between the anterior and vitreous
• Nonadministration of subconjunctival antibiotics
• Extra- or intracapsular cataract extraction*
• Other eye procedures during the same admission ( vitreoretinal procedure, lacrimal/eyelid procedures or adjunctive MMC for inferior filtration)
• Phacoemulsification
**Postoperative risk factors ( n = 6 )**
• Wound leak on the first postoperative day
• Topical antibiotic started the day after surgery
• Use of ciprofloxacin rather than ofloxacin topically after surgery
• Not patching after surgery
• Not placing a collagen shield soaked in antibiotic
• Length of stay ( ≥8 days )

*Results were put into meta-analysis.

### Inclusion and Exclusion Criteria

**Figure 1:**
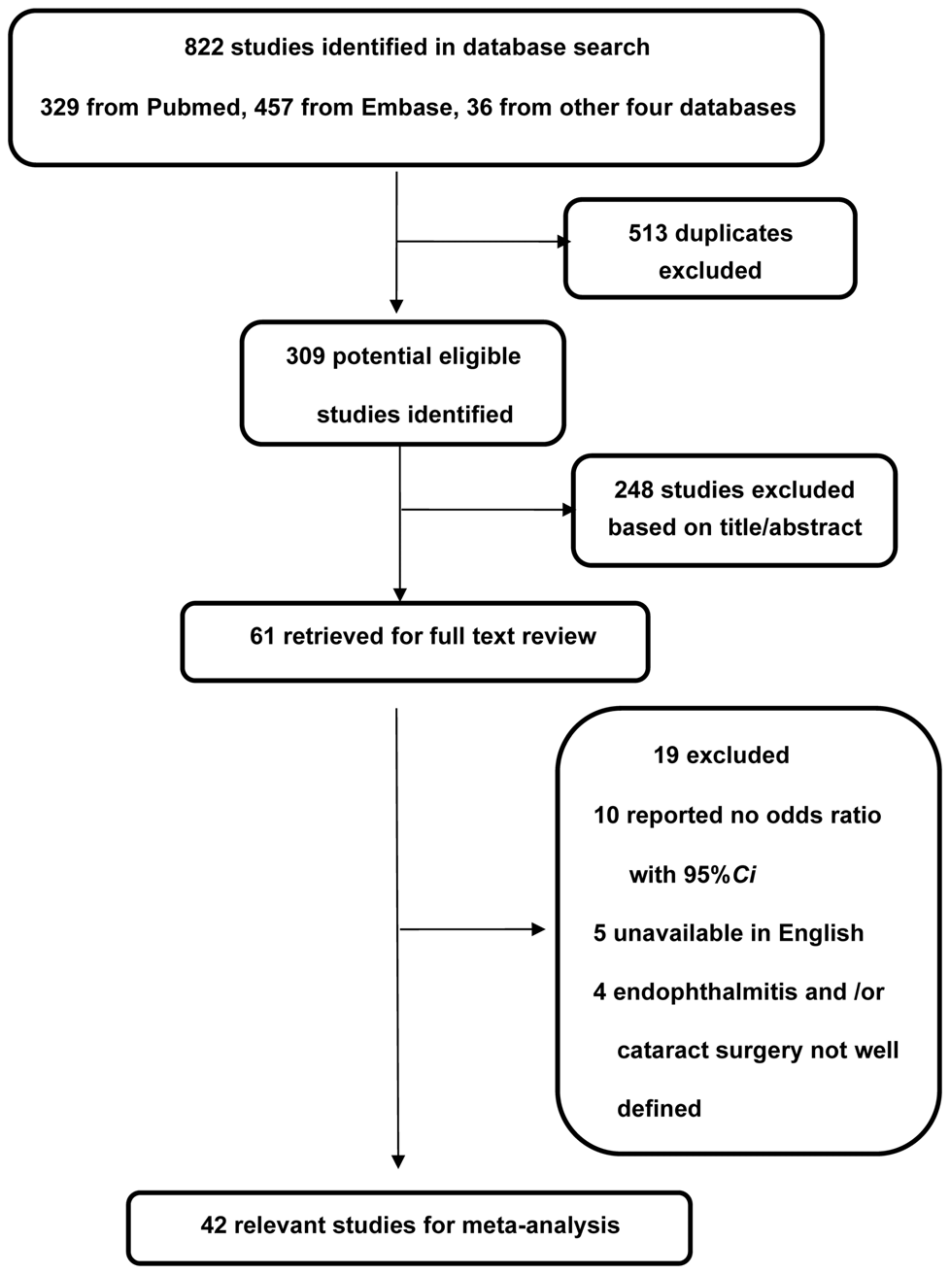
Flow diagram showing the selection process for inclusion of studies in the meta-analysis. CI  =  confidence interval.

Studies were included if they (i) reported cataract surgery as covariate, (ii) had exogenous acute endophthalmitis as the outcome measure, including the suspected and/or confirmed cases by positive culture, If a positive culture of a vitreous sample was obtained, we defined the case as a proven acute endophthalmitis. In all proven and unproven cases, the patients had swollen lids, pain and an opaque vitreous. (iii) reported a measure of the association either as odds ratio (OR) or relative risk (RR) with 95% confidence interval (CI), or allowed for the calculation of it from the raw data presented in the article, and (iv) the study examined human cases. We excluded (i) studies involving posttraumatic endophthalmitis or endogenous endophthalmitis, (ii) studies without a clear-cut definition of cataract surgery or detailed description of acute endophthalmitis assessment, (iii) samples including cases of TASS (toxic anterior segment syndrome), (iv) studies with underwent secondary lens implantation, intraocular lenses (IOLs) exchange, or cataract surgery combined with filtering procedures or corneal transplantation were excluded (Figure 1).

### Data Extraction and Quality Assessment

For each study, the following characteristics were extracted: (i) last name of first author, (ii) year of publication, (iii) study design and follow-up, (iv) location of the population, (v) date of the study, (vi) sample size and age range of subjects in the analysis, (vii) number of endophthalmitis, (viii) definition of cataract surgery, (ix) estimates of odds ratios (ORs), relative risks (RR), or the primary data required to calculate these ratios, (x) quality criteria. The clinical quality criteria was assessed with the Levels of Evidence offered by Oxford Centre for Evidence-based Medicine in March 2009 [12] (Table 2).

**Table 2:** Summary of the included studies evaluating risk factors and the association with acute endophthalmitis following cataract surgery.

Authors, Year [Ref]	Data Source	Study Design	Presentation interval(days)	Country	Date	Population Sample size, age[yrs]	Total Number of endophthalmitis (incidence rate%)	Definition of the endophthalmitis	Type of confirmed pathogen (n)	Risk factors (RR/OR) ^&^	Classification of cataract surgery	Quality Criteria^?^
Das T, 2012 [16]	ED	CC	15±12	India	1993∼ 1998	46 095 42∼81	62(0.13%)	Suspected (62) Confirmed (36)	S. Epidermidis(16); P. Aeruginosa(5); GPC (other)^#^ (8) Others(7)	OR: Ambulatory patients care (3.63) Residence location within city(2.27)	ECCE Phacoemulsification	B
Keay L, 2012 [17] ^?^	ED	RO	NR	USA	2003∼ 2004	3 280 966 NA	4 006 (0.012%)	Suspected (4006)	NR	RR: Male (1.23) Older individuals(≥85)(1.53) Black (1.17) Native Americans (1.72) Surgeons with low annual volume (3.8) Surgeons with less experience (1.41) surgeries performed in 2003 (1.2)	NA	B
Romero-Aroca P, 2012 [18] ^?^	ED	PO	5.37±2.33	Spain	1996∼ 2009	25 001 53∼89	83 (3.32%)	Suspected (83) Confirmed (55)	NR	RR: Without intracameral cefazolin (11.45)	Phacoemulsification	A
Tan CS, 2012 [19] ^?^	ED	Coh	NR	Singapore	11 years	50 177 NR	21(0.042%)	Suspected(21)	NR	OR: Without intracameral cefazolin (13.6) Age(1.05) Male gender(2.96)	NA	B
García-Sáenz MC 2010 [20] ^?^	ED	CS	NR	Spain	1999∼ 2009	15 173 NR	43(0.28%)	Suspected (43)	NR	RR: Without intracameral cefuroxime (8.57)	NA	A
Anijeet DR, 2010 [21]	ED	RO	1∼14	UK	1998∼ 2008	16 606 NR	14(0.078%)	Suspected(14) Confirmed(6)	GPC^#^(1); P. Streptococcus(3) P. aeruginosa (1); Nil(1)	RR: without intracameral vancomycin (38)	NA	B
Freeman EE 2010 [22] ^?^	ED	RO	<90	Canada	1996∼ 2005	490 690 NR	754(0.15%)	Suspected(754)	NR	OR:Older individuals(≥85)(1.34) Male gender(1.44) later year of surgery (0.94) Region of surgery 6 and 9(6∼9)	NA	B
Lloyd JC, 2009 [23]	ED	RO	1∼35	Canada	2006∼ 2005	13 931 NR	5(0.036%)	Suspected(5) Confirmed(4)	Staphylococcus(2); Streptococcus(1);P.acnes(1);	OR:Without fourth-generation fluoroquinolones perioperatively(6.3)	Phacoemulsification	B
Al-Mezaine HS, 2009 [25] ^?^	ED	RO	NR	Arabia	10	29 412 NR	20(0.068%)	Suspected(20) Confirmed(18)	Staphylococcus (7); Streptococcus(7); P.acnes(1); others(3)	OR:clear corneal incision(1.73)	ECCE Phacoemulsification	B
Garat M, 2009 [26] ^?^	ED	CC	NR	Spain	2002∼ 2007	18 579 NR	31(0.167%)	Suspected(27) Confirmed(4)	NR	RR: Without intracameral cefazolin(8.89)	Phacoemulsification	B
Hatch WV, 2009 [27] ^?^	ED	Coh	NR	Canada	2002∼ 2006	442 177 NR	617(0.14%)	Suspected(617)	NR	OR: Male gender(1.4) Capsular rupture(9.56) Older individuals(1.24)	ECCE Phacoemulsification	A
Pleyer U, 2008 [28] ^?^	ED	Coh	NR	Europe	NR	16211 NR	29(0.179%)	Suspected(29) Confirmed(20)	NR	RR: Without intracameral cefuroxime(4.92) Clear corneal incision(5.88) Silicon based IOL(3.13) Intraoperative complications(4.95)	Phacoemulsification	A
Garcia-Arumi J, 2007 [30] ^?^	ED	CC	<30	Spain	2002∼ 2003	5 011 NR	27(0.539%)	NR	NR	OR: Topical anesthesia(11.8) Surgery longer than 45minutes(7.2)	NA	A
Endophthalmitis Study Group, 2007 [31] ^?^	ED	Coh	NR	Austria, Belgium, Germany, Italy, etc.	NR	16 603 NR	29(0.175%)	Suspected(29) Confirmed(20)	NR	OR: Without intracameral cefuroxime (4.92) Clear corneal incision(5.88) Silicone IOL(3.13) Surgical complications(4.95) Male gender(2.70)	ECCE Phacoemulsification	A
Lundström M 2007 [32] ^?^	ED	PO	NR	Sweden	2002∼ 2004	225 471 NR	109(0.048%)	Suspected(109)	NR	RR: Communication between the anterior and vitreous Older individuals (≥85) Without intracameral cefuroxime	ECCE Phacoemulsification	A
Ng JQ, 2007 [33] ^?^	W	CC	NR	Australia	1980∼ 2000	1 025 NR	205(NA)	Suspected (205)	NR	RR: Same day surgery (2.27) Posterior capsule breach(13.57) Sub-conjunctival injection(0.46)	ECCE Phacoemulsification	B
Kamalarajah S, 2006 [34] ^?^	ED	CC	NR	UK	1999∼ 2000	659 (73.5–75)	214(NA)	Suspected (214)	NR	OR: Inpatient surgery(2.88) Dedicated center(2.76) Face masks not worn(3.34) Posterior capsule tear(3.82) Without subconjunctival antibiotics (6.85)	ECCE Phacoemulsification	A
Barry P, 2006 [35] ^?^	ED	Coh	NR	Austria, Belgium, Germany, Italy, etc.	2003∼ 2006	13 698 NR	28(0.2%)	Suspected (28)	NR	OR: Without intracameral cefuroxime (4.59)	Phacoemulsification	A
Wu PC, 2006 [36]	ED	CC	NR	Taiwan	1992∼ 2000	10 614 NR	12(0.113%)	Suspected (12)	NR	OR: skin preparation with 5% povidone-iodine(10.9) without 5% povidone-iodine conjunctival disinfection(5.6)	ECCE	B
Wejde G, 2005 [37] ^?^	ED	CC	NR	Sweden	1994∼ 2000	44 986 NR	60(0.133%)	Suspected(60)	NR	RR: without intracameral cefuroxime(5.7) ECCE/ICCE(2.1) silicone lens((3.4)	ECCE Phacoemulsification	B
Lalitha P, 2005 [38] ^?^	ED	RO	NR	India	2002∼ 2003	36 072 NR	19(0.053%)	Suspected(10) Confirmed(9)	NR	RR: posterior capsular rent(6.57) ECCE(4.9) older individuals(6.0)	ECCE Phacoemulsification	B
West ES, 2005 [39] ^?^	ED	RO	NR	USA	1994∼ 2001	477 627 NR	1 026(0.215%)	Suspected(10) Confirmed(9)	NR	RR: surgeries before 1998(1.44) old individuals(90)(1.83) black race(1.30)	ECCE Phacoemulsification	A
Wallin T, 2005 [40] ^?^	ED	Coh	NR	USA	1996∼ 2002	1552 NR	27(1.7%)	Suspected(27)	NR	RR: Wound leak(44) Capsular complication(17.2) Topical antibiotic after surgery(13.7) With ciprofloxacin (5.3) Without patching (7.1) Without collagen shield (2.7)	ECCE Phacoemulsification	B
Wejde G, 2005 [41] ^?^	ED	PO	NR	Sweden	1999∼ 2001	188 151 NR	109(0.0595%)	Suspected (91) Confirmed (21)	GPB^#^	RR: Without intracameral cefuroxime (3.649) ECCE/ICCE (1.7)	ECCE Phacoemulsification	A
Li J, 2004 [42] ^?^	W	PO	NR	Australia	1980∼ 2000	117 083 NR	210(0.179%)	Suspected (210)	NR	OR: Male 0.89(0.67–1.19) Older individuals(>80)1.50(1.13–1.99) Length of stay(+8days) (2.08) Private hospital (2.38) Length of study (>8days)(2.08) With vitreoretinal procedure (2.71) Lacrimal/eyelid procedures (23.5)	ECCE ICCE Phacoemulsification other cataract extraction/ lens-related procedures	A
Wong TY, 2004 [43] ^?^	ED	PO	NR	Singapore	1996∼ 2001	44 803 NR	34(0.076%)	Suspected (34) Confirmed (21)		RR:Phacoemulsification (3.1) Posterior capsule rupture (8.0)	ECCE Phacoemulsification	A
Wong TY, 2004 [44] ^?^	ED	CC	54∼80	Singapore Chinese, Malay,etc.	1996∼ 2001	136 NR	34(NA)	Suspected (34) Confirmed (21)	Staphylococcus (13) Streptococci (1); P.aeroginosa(1) Others(6)	OR: Silicone IOL (5.1) Posterior capsular rupture (20.9)	ECCE Phacoemulsification	B
Cooper BA, 2003 [45] ^?^	ED	CC	NR	USA	1997∼ 2000	409 NR	38(NA)	Confirmed(38)	NR	OR: Clear corneal incision (3.36)	ECCE Phacoemulsification	B
E Mayer, 2003 [46] ^?^	ED	RO	NR	UK	1991∼ 2001	18 191 (43–89)	30(0.16%)	Suspected (30) Confirmed (26)	Staphylococcus (9) P. Streptococcus (2) P.aeruginosa (1) Others(14)	RR: ECCE (4.37) Folded IOL (43.8) Capsular rupture (2.06)	ECCE Phacoemulsification	B
Ellis MF, 2003 [47] ^?^	ED	RO	NR	Australia	1997∼ 2001	633 NR	5(0.78%)	Confirmed (5)	S. epidermidis (5)	OR: Topical anesthesia (7.63)	Phacoemulsification	B
Nagaki Y, 2003 [48] ^?^	ED	PO	NR	Japan	1998∼ 2001	11595 NR	16(0.14%)	Suspected (16) Confirmed (13)	NR	RR: Clear corneal incision (5.61)	Phacoemulsification	A
Kalpadakis P, 2002 [49] ^?^	ED	RO	NR	Greece	1998∼ 2001	2 446 NR	20(0.82%)	Suspected (20)	NR	RR: ECCE (1.99)	ECCE Phacoemulsification	A
Montan P, 2002 [50] ^?^	ED	PO	NR	Sweden	1998	54 666 NR	58(0.106%)	Suspected (58) Confirmed (41)	CNS ^#^(15) Streptococci (12) S. aureus(4)	OR: PMMA (2.03) Silicon (1.93) Hydrogel (2.89)	Phaco + PC IOL ECCE + PC IOL Phaco/ECCE + AC IOL	A
Lertsumitkul S, 2001[51] ^?^	W	CC	NR	Australia	1996∼ 1998	97 NR	31(NA)	Suspected (31) Confirmed (20)	S. Epidermidis(9) S. aureus(5) Streptococcus(2)	OR: Surgical complications (6.34) Clear corneal temporal incision (3.52)	ECCE Phacoemulsification Penetrating keratoplasty	B
Colleaux KM, 2000 [52]	ED	RO	NR	Sask	1994∼ 1998	13 886 NR	25 (0.18%)	Suspected (25)	NR	OR: without subconjunctival antibiotic injections (16.23)	Phacoemulsification	B
Swaddiwudhipong W, 2000 [53] ^?^	ED	RO	NR	Thailand	1997∼ 1998	329 NR	31(9.4%)	Suspected (31)	NR	RR: ECCE (0.47)	Phacoemulsification145 ECCE165 secondary IOL implantation	B
Schmitz S, 1999 [54]	ED	CS	NR	Germany	1996	340 633 NR	267 (0.148%)	NR	NR	OR: preoperative topical antibiotics (2.38) outpatient surgeries (2.0)	NA	A
Bainbridge JW, 1998 [55] ^?^	ED	RO	12∼101	United Kingdom	NA	772 NR	7(0.91%)	Suspected (7) Confirmed(5)	CNS^#^ (3) S. aureus(1)	RR: Silicone IOL (20.73)	Phacoemulsification	B
Norregaard JC, 1997 [56] ^?^	ED	RO	NR	Denmark	1985∼ 1987	19 246 NR	61(0.317%)	Suspected (61)	NR	OR: Old individuals≥90 (3.62) Male gender (1.93) ICCE (2.22) Capsular rupture (4.86)	ECCE ± IOL ICCE ± IOL	B
Javitt JC, 1991 [57] ^?^	ED	RO	NR	USA	1984	324 032 NR	439(0.135%)	Suspected(439)	NR	OR: Capsular rupture (4.56) Male gender (1.23)	ICCE 99971 ECCE 195587 Phacoemulsification 28474	B
Authors, Year [Ref]	Data Source	Study Design	Presentation interval(days)	Country	Date	Population Sample size, age[yrs]	Total Number of endophthalmitis (incidence rate%)	Definition of the endophthalmitis	Type of confirmed pathogen (n)	Risk factors (RR/OR) ^&^	Classification of cataract surgery	Quality Criteria^?^
Das T, 2012 [16]	ED	CC	15±12	India	1993∼ 1998	46 095 42∼81	62(0.13%)	Suspected (62) Confirmed (36)	S. Epidermidis(16); P. Aeruginosa(5); GPC (other)^#^ (8) Others(7)	OR: Ambulatory patients care (3.63) Residence location within city(2.27)	ECCE Phacoemulsification	B
Keay L, 2012 [17] ^?^	ED	RO	NR	USA	2003∼ 2004	3 280 966 NA	4 006 (0.012%)	Suspected (4006)	NR	RR: Male (1.23) Older individuals(≥85)(1.53) Black (1.17) Native Americans (1.72) Surgeons with low annual volume (3.8) Surgeons with less experience (1.41) surgeries performed in 2003 (1.2)	NA	B
Romero-Aroca P, 2012 [18] ^?^	ED	PO	5.37±2.33	Spain	1996∼ 2009	25 001 53∼89	83 (3.32%)	Suspected (83) Confirmed (55)	NR	RR: Without intracameral cefazolin (11.45)	Phacoemulsification	A
Tan CS, 2012 [19] ^?^	ED	Coh	NR	Singapore	11 years	50 177 NR	21(0.042%)	Suspected(21)	NR	OR: Without intracameral cefazolin (13.6) Age(1.05) Male gender(2.96)	NA	B
García-Sáenz MC 2010 [20] ^?^	ED	CS	NR	Spain	1999∼ 2009	15 173 NR	43(0.28%)	Suspected (43)	NR	RR: Without intracameral cefuroxime (8.57)	NA	A
Anijeet DR, 2010 [21]	ED	RO	1∼14	UK	1998∼ 2008	16 606 NR	14(0.078%)	Suspected(14) Confirmed(6)	GPC^#^(1); P. Streptococcus(3) P. aeruginosa (1); Nil(1)	RR: without intracameral vancomycin (38)	NA	B
Freeman EE 2010 [22] ^?^	ED	RO	<90	Canada	1996∼ 2005	490 690 NR	754(0.15%)	Suspected(754)	NR	OR:Older individuals(≥85)(1.34) Male gender(1.44) later year of surgery (0.94) Region of surgery 6 and 9(6∼9)	NA	B
Lloyd JC, 2009 [23]	ED	RO	1∼35	Canada	2006∼ 2005	13 931 NR	5(0.036%)	Suspected(5) Confirmed(4)	Staphylococcus(2); Streptococcus(1);P.acnes(1);	OR:Without fourth-generation fluoroquinolones perioperatively(6.3)	Phacoemulsification	B
Al-Mezaine HS, 2009 [25] ^?^	ED	RO	NR	Arabia	10	29 412 NR	20(0.068%)	Suspected(20) Confirmed(18)	Staphylococcus (7); Streptococcus(7); P.acnes(1); others(3)	OR:clear corneal incision(1.73)	ECCE Phacoemulsification	B
Garat M, 2009 [26] ^?^	ED	CC	NR	Spain	2002∼ 2007	18 579 NR	31(0.167%)	Suspected(27) Confirmed(4)	NR	RR: Without intracameral cefazolin(8.89)	Phacoemulsification	B
Hatch WV, 2009 [27] ^?^	ED	Coh	NR	Canada	2002∼ 2006	442 177 NR	617(0.14%)	Suspected(617)	NR	OR: Male gender(1.4) Capsular rupture(9.56) Older individuals(1.24)	ECCE Phacoemulsification	A
Pleyer U, 2008 [28] ^?^	ED	Coh	NR	Europe	NR	16211 NR	29(0.179%)	Suspected(29) Confirmed(20)	NR	RR: Without intracameral cefuroxime(4.92) Clear corneal incision(5.88) Silicon based IOL(3.13) Intraoperative complications(4.95)	Phacoemulsification	A
Garcia-Arumi J, 2007 [30] ^?^	ED	CC	<30	Spain	2002∼ 2003	5 011 NR	27(0.539%)	NR	NR	OR: Topical anesthesia(11.8) Surgery longer than 45minutes(7.2)	NA	A
Endophthalmitis Study Group, 2007 [31] ^?^	ED	Coh	NR	Austria, Belgium, Germany, Italy, etc.	NR	16 603 NR	29(0.175%)	Suspected(29) Confirmed(20)	NR	OR: Without intracameral cefuroxime (4.92) Clear corneal incision(5.88) Silicone IOL(3.13) Surgical complications(4.95) Male gender(2.70)	ECCE Phacoemulsification	A
Lundström M 2007 [32] ^?^	ED	PO	NR	Sweden	2002∼ 2004	225 471 NR	109(0.048%)	Suspected(109)	NR	RR: Communication between the anterior and vitreous Older individuals (≥85) Without intracameral cefuroxime	ECCE Phacoemulsification	A
Ng JQ, 2007 [33] ^?^	W	CC	NR	Australia	1980∼ 2000	1 025 NR	205(NA)	Suspected (205)	NR	RR: Same day surgery (2.27) Posterior capsule breach(13.57) Sub-conjunctival injection(0.46)	ECCE Phacoemulsification	B
Kamalarajah S, 2006 [34] ^?^	ED	CC	NR	UK	1999∼ 2000	659 (73.5–75)	214(NA)	Suspected (214)	NR	OR: Inpatient surgery(2.88) Dedicated center(2.76) Face masks not worn(3.34) Posterior capsule tear(3.82) Without subconjunctival antibiotics (6.85)	ECCE Phacoemulsification	A
Barry P, 2006 [35] ^?^	ED	Coh	NR	Austria, Belgium, Germany, Italy, etc.	2003∼ 2006	13 698 NR	28(0.2%)	Suspected (28)	NR	OR: Without intracameral cefuroxime (4.59)	Phacoemulsification	A
Wu PC, 2006 [36]	ED	CC	NR	Taiwan	1992∼ 2000	10 614 NR	12(0.113%)	Suspected (12)	NR	OR: skin preparation with 5% povidone-iodine(10.9) without 5% povidone-iodine conjunctival disinfection(5.6)	ECCE	B
Wejde G, 2005 [37] ^?^	ED	CC	NR	Sweden	1994∼ 2000	44 986 NR	60(0.133%)	Suspected(60)	NR	RR: without intracameral cefuroxime(5.7) ECCE/ICCE(2.1) silicone lens((3.4)	ECCE Phacoemulsification	B
Lalitha P, 2005 [38] ^?^	ED	RO	NR	India	2002∼ 2003	36 072 NR	19(0.053%)	Suspected(10) Confirmed(9)	NR	RR: posterior capsular rent(6.57) ECCE(4.9) older individuals(6.0)	ECCE Phacoemulsification	B
West ES, 2005 [39] ^?^	ED	RO	NR	USA	1994∼ 2001	477 627 NR	1 026(0.215%)	Suspected(10) Confirmed(9)	NR	RR: surgeries before 1998(1.44) old individuals(90)(1.83) black race(1.30)	ECCE Phacoemulsification	A
Wallin T, 2005 [40] ^?^	ED	Coh	NR	USA	1996∼ 2002	1552 NR	27(1.7%)	Suspected(27)	NR	RR: Wound leak(44) Capsular complication(17.2) Topical antibiotic after surgery(13.7) With ciprofloxacin (5.3) Without patching (7.1) Without collagen shield (2.7)	ECCE Phacoemulsification	B
Wejde G, 2005 [41] ^?^	ED	PO	NR	Sweden	1999∼ 2001	188 151 NR	109(0.0595%)	Suspected (91) Confirmed (21)	GPB^#^	RR: Without intracameral cefuroxime (3.649) ECCE/ICCE (1.7)	ECCE Phacoemulsification	A
Li J, 2004 [42] ^?^	W	PO	NR	Australia	1980∼ 2000	117 083 NR	210(0.179%)	Suspected (210)	NR	OR: Male 0.89(0.67–1.19) Older individuals(>80)1.50(1.13–1.99) Length of stay(+8days) (2.08) Private hospital (2.38) Length of study (>8days)(2.08) With vitreoretinal procedure (2.71) Lacrimal/eyelid procedures (23.5)	ECCE ICCE Phacoemulsification other cataract extraction/ lens-related procedures	A
Wong TY, 2004 [43] ^?^	ED	PO	NR	Singapore	1996∼ 2001	44 803 NR	34(0.076%)	Suspected (34) Confirmed (21)		RR:Phacoemulsification (3.1) Posterior capsule rupture (8.0)	ECCE Phacoemulsification	A
Wong TY, 2004 [44] ^?^	ED	CC	54∼80	Singapore Chinese, Malay,etc.	1996∼ 2001	136 NR	34(NA)	Suspected (34) Confirmed (21)	Staphylococcus (13) Streptococci (1); P.aeroginosa(1) Others(6)	OR: Silicone IOL (5.1) Posterior capsular rupture (20.9)	ECCE Phacoemulsification	B
Cooper BA, 2003 [45] ^?^	ED	CC	NR	USA	1997∼ 2000	409 NR	38(NA)	Confirmed(38)	NR	OR: Clear corneal incision (3.36)	ECCE Phacoemulsification	B
E Mayer, 2003 [46] ^?^	ED	RO	NR	UK	1991∼ 2001	18 191 (43–89)	30(0.16%)	Suspected (30) Confirmed (26)	Staphylococcus (9) P. Streptococcus (2) P.aeruginosa (1) Others(14)	RR: ECCE (4.37) Folded IOL (43.8) Capsular rupture (2.06)	ECCE Phacoemulsification	B
Ellis MF, 2003 [47] ^?^	ED	RO	NR	Australia	1997∼ 2001	633 NR	5(0.78%)	Confirmed (5)	S. epidermidis (5)	OR: Topical anesthesia (7.63)	Phacoemulsification	B
Nagaki Y, 2003 [48] ^?^	ED	PO	NR	Japan	1998∼ 2001	11595 NR	16(0.14%)	Suspected (16) Confirmed (13)	NR	RR: Clear corneal incision (5.61)	Phacoemulsification	A
Kalpadakis P, 2002 [49] ^?^	ED	RO	NR	Greece	1998∼ 2001	2 446 NR	20(0.82%)	Suspected (20)	NR	RR: ECCE (1.99)	ECCE Phacoemulsification	A
Montan P, 2002 [50] ^?^	ED	PO	NR	Sweden	1998	54 666 NR	58(0.106%)	Suspected (58) Confirmed (41)	CNS ^#^(15) Streptococci (12) S. aureus(4)	OR: PMMA (2.03) Silicon (1.93) Hydrogel (2.89)	Phaco + PC IOL ECCE + PC IOL Phaco/ECCE + AC IOL	A
Lertsumitkul S, 2001[51] ^?^	W	CC	NR	Australia	1996∼ 1998	97 NR	31(NA)	Suspected (31) Confirmed (20)	S. Epidermidis(9) S. aureus(5) Streptococcus(2)	OR: Surgical complications (6.34) Clear corneal temporal incision (3.52)	ECCE Phacoemulsification Penetrating keratoplasty	B
Colleaux KM, 2000 [52]	ED	RO	NR	Sask	1994∼ 1998	13 886 NR	25 (0.18%)	Suspected (25)	NR	OR: without subconjunctival antibiotic injections (16.23)	Phacoemulsification	B
Swaddiwudhipong W, 2000 [53] ^?^	ED	RO	NR	Thailand	1997∼ 1998	329 NR	31(9.4%)	Suspected (31)	NR	RR: ECCE (0.47)	Phacoemulsification145 ECCE165 secondary IOL implantation	B
Schmitz S, 1999 [54]	ED	CS	NR	Germany	1996	340 633 NR	267 (0.148%)	NR	NR	OR: preoperative topical antibiotics (2.38) outpatient surgeries (2.0)	NA	A
Bainbridge JW, 1998 [55] ^?^	ED	RO	12∼101	United Kingdom	NA	772 NR	7(0.91%)	Suspected (7) Confirmed(5)	CNS^#^ (3) S. aureus(1)	RR: Silicone IOL (20.73)	Phacoemulsification	B
Norregaard JC, 1997 [56] ^?^	ED	RO	NR	Denmark	1985∼ 1987	19 246 NR	61(0.317%)	Suspected (61)	NR	OR: Old individuals≥90 (3.62) Male gender (1.93) ICCE (2.22) Capsular rupture (4.86)	ECCE ± IOL ICCE ± IOL	B
Javitt JC, 1991 [57] ^?^	ED	RO	NR	USA	1984	324 032 NR	439(0.135%)	Suspected(439)	NR	OR: Capsular rupture (4.56) Male gender (1.23)	ICCE 99971 ECCE 195587 Phacoemulsification 28474	B

Note: ED, electronic database; W, Internet search; CC, case-control; Coh, cohort; CS, cross-sectional; RO, retrospective observational; PO, prospective observational; PK, Penetrating keratoplasty; ECCE, extracapsular cataract extraction; ICCE, intracapsular extraction; AC IOL: anterior chamber intraocular lens; PC IOL: posterior chamber intraocular lens; NA, not available; NR, not recorded; N, domain not addressed. *Results were put into meta-analysis & OR/RR with 95% confidence interval is for all suspected endophthalmitis cases, not limited the confirmed (culture-positive) endophthalmitis. # GPC: Gram-positive cocci other than S. epidermidis. GNB: Gram-negative bacilli other than P. aeruginosa. GPB: Gram-positive bacilli other than P. Acnes. CNS*: Coagulase negative staphylococci; ?The quality criteria: A  =  consistent level 1 studies (1a-c), B  =  consistent level 2 or 3 studies or extrapolations from level 1 studies(2a–c,3a–b) 1a,SR (with homogeneity) of RCTs; 1b: Individual RCT (with narrow Confidence Interval);1c: all or none; 2a: SR (with homogeneity) of cohort studies;2b: Individual cohort study (including low quality RCT; e.g., <80% follow-up);2c: “Outcomes” Research; Ecological studies;3a: SR (with homogeneity*) of case-control studies;3b: Individual Case-Control Study. All the included studies have no conflict of Interest.

### Statistical Analysis

The fully adjusted, study-specific ORs or RR were combined to estimate the pooled OR with 95% CI using the random effects model. RR was treated as ORs without further adjustment as the incidence of acute endophthalmitis in the studied populations was low (ie, <10%) [77]. Most of the studies included in our meta-analysis reported both an OR for any risk factor and ORs after stratification (Table 3). The random effects model was chosen because it accounts for both within-study and between-study variability. As our expect lies on testing for eventual differences between coefficients for the random effects model and the fixed effects model, we use a generalized Hausman specification test. The advantage of such specification test is that it makes use of the sandwich covariance estimator to adjust for any heteroskedasticity in the outcomes. For comparative purposes, we followed the same model for estimating the summary results of each factor.

**Table 3:** Summary results from the meta-analysis.

Risk factor				Prospective		Cross sectional		Retrospective
	n	Pooled odds ratio	n	Overall estimate	n		n	Overall estimate
Male gender	6	1.41(1.22∼1.63)	1	2.70(1.07∼6.80)	0	NA	5	1.38(1.21∼1.58)
Older individuals (≥85 years)	7	1.50(1.18∼1.91)	0	NA	0	NA	7	1.50(1.18∼1.91)
Without intracameral cefazolin	3	10.76(6.45∼17.95)	1	11.45(5.73∼22.88)	0	NA	2	9.97(4.66∼21.33)
Without intracameral cefuroxime	6	5.48(3.79∼7.92)	4	4.09(2.86∼5.84)	1	8.55(6.21∼11.76)	1	5.70(2.77∼11.75)
Posterior capsular rupture	10	6.34(4.23∼9.52)	1	3.82(1.67∼8.72)	0	NA	9	6.82(4.41∼10.55)
Clear corneal incision	6	3.60(2.05∼6.31)	3	5.65(3.75∼8.52)	0	NA	3	2.44(1.46∼4.09)
Silicone based IOL	6	3.02(2.03∼4.49)	3	2.35(1.67∼3.30)	0	NA	3	4.64(2.25∼9.56)
Intraoperative complications	3	5.29(2.73∼10.18)	2	4.95(2.31∼10.63)	0	NA	1	6.34(1.77∼22.67)
ECCE/ICCE	6	2.19(1.40∼3.24)	0	NA	0	NA	6	2.19(1.40∼3.24)

n  =  number of estimates entered in the models; NA: not available.

Funnel plots were reviewed for each risk factors and no evidence of publication bias was observed.

Statistical heterogeneity among studies was evaluated using I^2^ Statistic. I^2^ is the percentage of the total variation across the studies that is due to heterogeneity [13]. Values of ≤24%, 25% to 49%, 50% to 74%, and >75% denote no, low, moderate, and high heterogeneity, respectively [14]. Heterogeneity due to study design was avoided by restricting the main analyses to the same study design respectively. Furthermore, we performed a sensitivity analysis that investigates the contribution of each study to the heterogeneity by sequentially omitting one study and reanalyzing the pooled estimate for the remaining studies [15]. Publication bias was evaluated with the use of Egger regression asymmetry test and the Begg's test. All statistical analyses were performed with Stata version 11.1 (StataCorp, College Station, TX). A 2-sided P value less than 0.05 was regarded as significant for all analyses.

## Results

In total, data from 10 prospective studies [18], [28], [31]–[32], [34]–[35], [41], [43], [48], [50], 2 cross-sectional studies [20], [54] and 30 retrospective studies [16 ∼ 17, 19, 21 ∼ 27, 29 ∼ 30, 33, 36 ∼ 40, 42, 44 ∼ 47, 49, 51 ∼ 53, 55 ∼ 57] were included in the final analysis, contributing a sample of some 6 686 169 patients including 8 963 endophthalmitis cases. Table 2 summarizes the characteristics of the concluded studies from which estimates were included in the meta-analysis. Funnel plots were reviewed for each risk factors and no evidence of publication bias was observed. The findings for each risk factor were summarized in Table 3 and discussed separately in the following sections.

### Age

**Figure 2:**
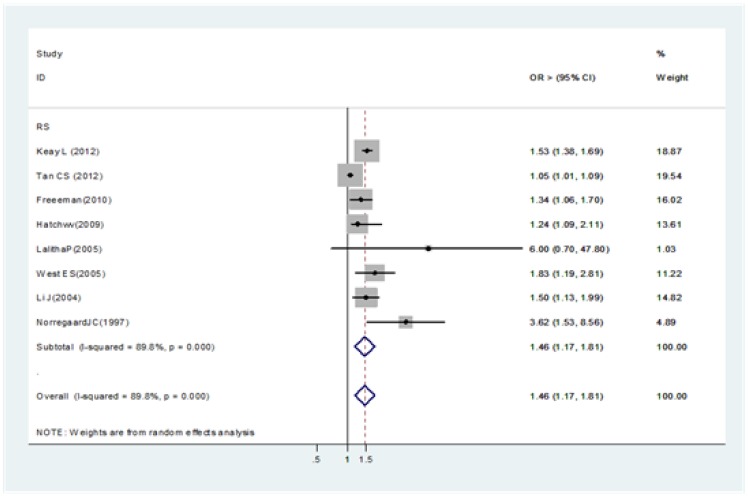
Pooled odds ratio for acute endophthalmitis and by age (≥85 years vs.<85 years).

All studies found a strong association with older individuals (≥85 years) (Figure 2) [17, 19 , 22, 27, 38∼39, 42, 56].

### Male gender

**Figure 3:**
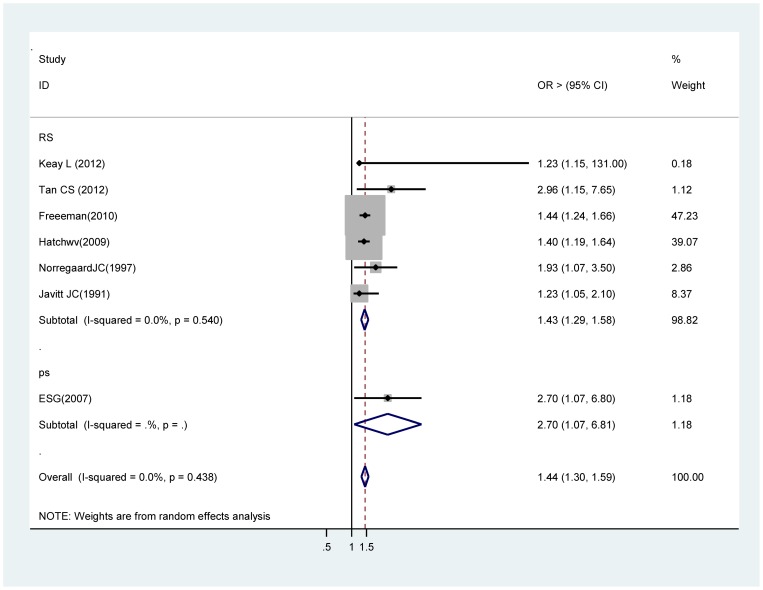
Pooled odds ratio for acute endophthalmitis by gender (male vs. female).

Six estimates from retrospective studies [17], [19], [22], [27], [56], [57] , and one prospective studies [31] contributed to this meta-analysis. Findings from this analysis suggest that there is a significant association between male gender and acute endophthalmitis. In the retrospective studies, the overall OR for male gender was 1.43 (95% CI 1.29 ∼ 1.58) , in the whole studies, it was 1.44 (95% CI 1.30 ∼ 1. 59) (Figure 3).

### Extra- or intracapsular cataract extraction (ECCE / ICCE)

**Figure 4:**
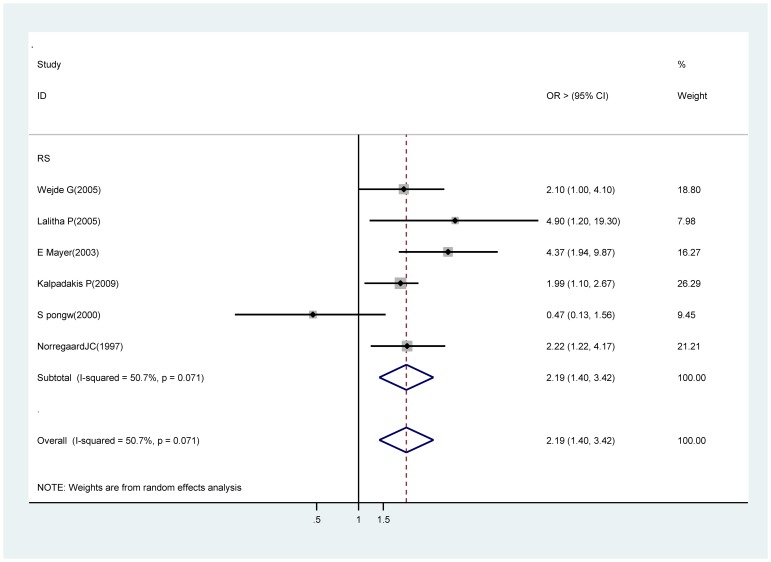
Pooled odds ratio for acute endophthalmitis by extra- or intracapsular cataract extraction (vs. phacoemulsification).

Six retrospective studies were included in the meta-analysis [37∼38, 46, 49, 53, 56]. Findings from the meta-analysis show an significant association between extra- or intracapsular cataract extraction and acute endophthalmitis (OR 2.19, 95% CI 1.40∼3.42) compared with phacoemulsification (Figure 4). Sensitivity analysis showed that the Swaddiwudhi pong W study [53] substantially influenced the pooled OR. After excluding this studies, the pooled OR was 2.37 (95% CI, 1.77∼3.17) with no evidence of heterogeneity (I^2^ = 0%; *P* = 0.41).

### A clear corneal incision

**Figure 5:**
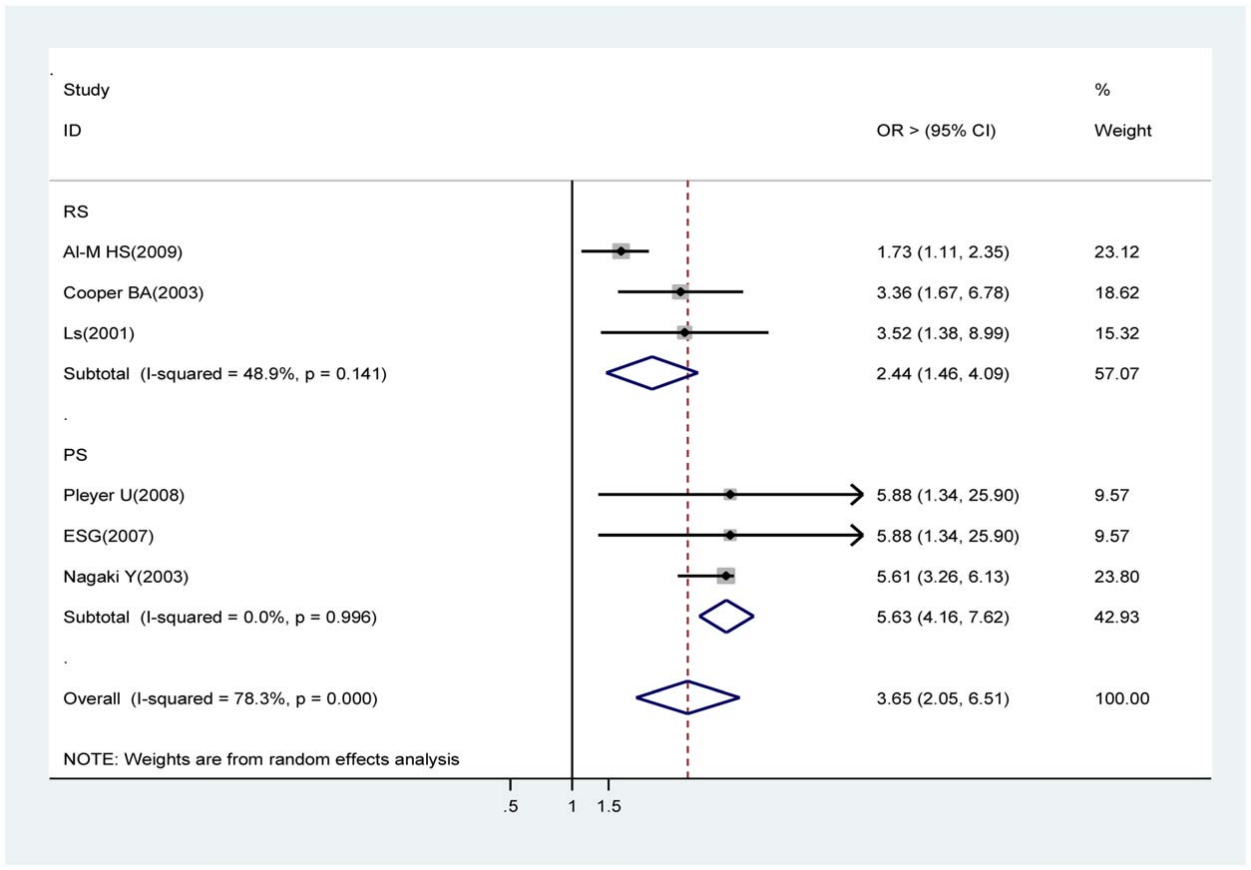
Pooled odds ratio for acute endophthalmitis by a clear corneal incision (vs. sclerocorneal incision or scleral tunnel incision).

Estimates from three prospective cohort studies ( i.e., Pleyer U, Endophthalmitis Study Group, and Nagaki Y) [28], [31], [48] and from three retrospective studies (Al-Mezaine HS, Cooper BA, Lertsumitkol S) [25], [45], [51], were analyzed. Analysis of the prospective cohort studies showed that a clear corneal incision is a strong risk factor for acute endophthalmitis (OR 5.65, 95% CI 3.75∼8.52). This finding is supported by the results of the meta-analysis of the retrospective studies (OR 2.44, 95% CI 1.46∼4.09) (Figure 5).

### Without intracameral cefazolin (1 mg in 0.1 ml solution)

**Figure 6:**
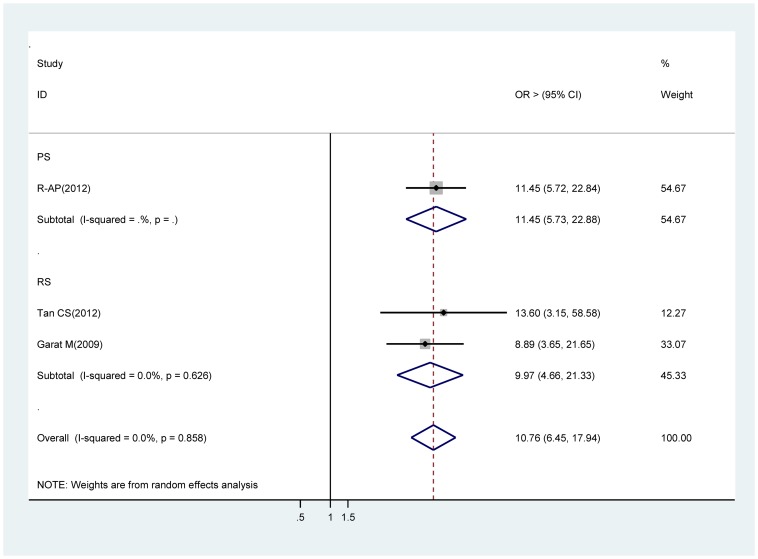
Pooled odds ratio for acute endophthalmitis by without intracameral cefazolin.

Estimates were reported from one prospective cohort studies [18] and two case retrospective studies [19], [26] contributed to the meta-analysis. Significant increases in acute endophthalmitis risk were seen in all the meta-analyses for without intracameral cefazolin during cataract surgery procedure. The OR for prospective studies was 11.45 (95% CI 5.72∼22.84), and that from retrospective studies was 9.97 (95% CI 4.66∼21.33). The OR obtained through analysis of the whole studies was 10.76 (95% CI 6.45∼17.94) (Figure 6).

### Without intracameral cefuroxime (1 mg in 0.1 ml solution)

**Figure 7:**
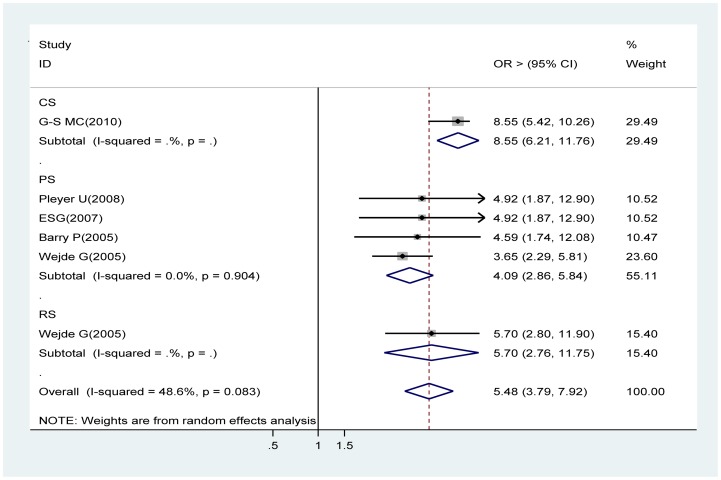
Pooled odds ratio for acute endophthalmitis by without intracameral cefuroxime.

Data from four prospective cohort studies [28], [31], [35], [41], one retrospective studies [37] and a pooled estimate from a cross sectional study [20] were used in the meta-analysis. The meta-analysis of the prospective cohort and retrospective studies suggests that intracameral cefuroxime is the protective effect against acute endophthalmitis compared with topical anti-infectives alone (OR 4.09, 95% CI 2.86∼5.84 , OR 5.70, 95% CI 2.76∼11.75 respectively). The overall OR with the cross sectional study supported this finding (OR 5.48, 95% CI 3.79∼7.92) (Figure 7).

### Posterior capsular rupture (PCR)

**Figure 8:**
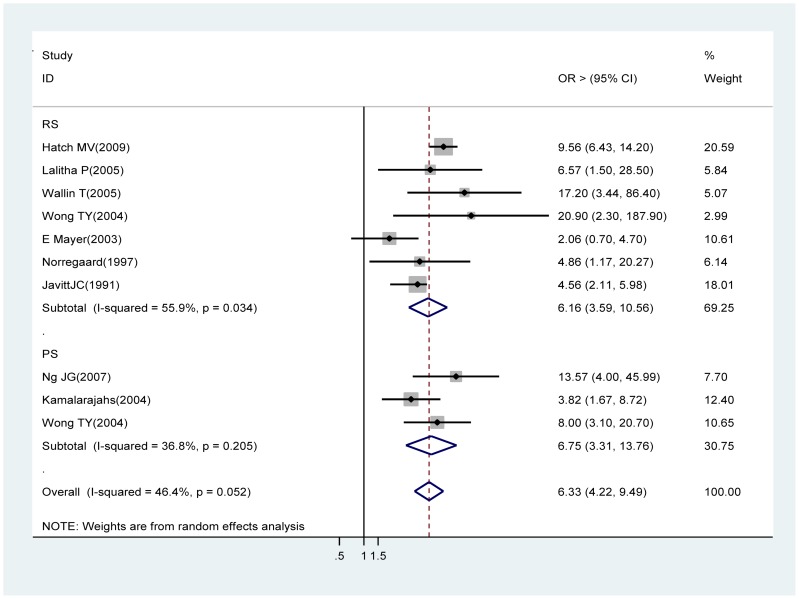
Pooled odds ratio for acute endophthalmitis by posterior capsular rupture.

Eight retrospective studies [27, 33, 38, 40, 44, 46, 56∼57] and two prospective studies [34], [43] contributed to the meta-analysis. Analysis of the retrospective studies showed PCR, a common complication of cataract surgery, was also a significant risk factor of acute endophthalmitis (OR 6.33, 95% CI 4.22∼9.49). Prospective study findings were in the same direction (OR 6.75, 95% CI 3.31∼13.76) (Figure 8).

### Silicone IOLs

**Figure 9:**
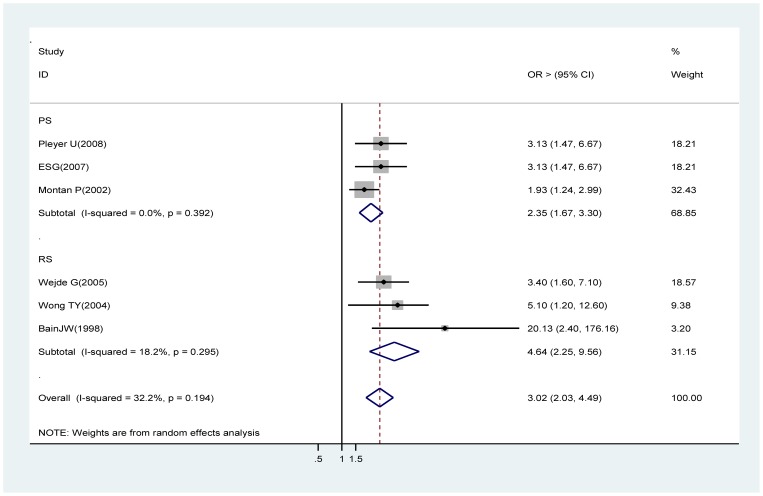
Pooled odds ratio for acute endophthalmitis by silicone based IOLs (compared with PMMA or acrylic IOLs).

Estimates from three prospective cohort studies [28], [31], [50] and from three retrospective studies [37], [44], [55] contributed to this analysis. Both of the analyses showed statistically significant associations (prospective cohort studies OR 2.35, 95% CI 1.67∼3.30; retrospective studies OR 4.64, 95% CI: 2.25∼9.56). The whole studies did identify a significant association between silicone intraocular lens and acute endophthalmitis (OR 3.02, 95% CI 2.03∼4.49) (Figure 9).

### Intraoperative complications

**Figure 10:**
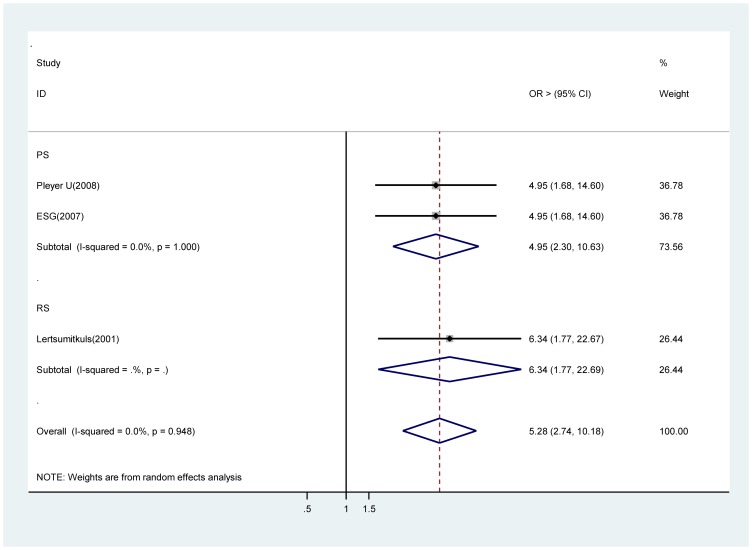
Pooled odds ratio for acute endophthalmitis by intraoperative complications.

A significant increase in risk of acute endophthalmitis with intraoperative complications in two prospective studies [28 ∼ 31], (OR 4.95, 95% CI 2.31∼10.63). There was only one estimate from a retrospective study [51] and it also supported this finding (OR 6.34, 95% CI 1.77∼22.67) of significance (Figure 10).

## Discussion

Identifying patients at high risk of acute endophthalmitis after cataract surgery is important from both public health and clinical perspectives as this would facilitate detection of disease before the onset of irreversible visual loss enabling earlier intervention. Of the nine risk factors identified in our systematic review and meta-analysis, ECCE / ICCE, a clear corneal incision, without intracameral cefazolin (1 mg in 0.1 ml solution) , without intracameral cefuroxime (1 mg in 0.1 ml solution), PCR, silicone intraocular lens and intraoperative complications were strongly and consistently associated with acute endophthalmiytis. All of these are easily assessed through discussions with patients and do not entail a lengthy medical history taking or laboratory evaluations. Other significant factors with a lower strength of association (risk estimates generally 1.5 or less) were male gender and old individuals (85 years and older). All of these factors are likely to be measured and monitored in the primary care setting.

As we and others have previously reported, we found that patient factors such as older age and male gender are associated with a higher risk of endophthalmitis after cataract surgery. The increased risk with age was only true for the very old ages (85 years) and this result might be explained by a reduced natural immunity in this advanced age group [22], [32], [42]. Several studies have reported increased rates of adverse postsurgical events among men [22], [31]. Using the analysis data, researchers noted that men had 41% higher odds of postoperative endophthalmitis, compared with women. Possible explanations for the higher complication rates in male patients include behavioral differences (e.g., adherence to postoperative instructions and antibiotic use) [58]; differences in bacterial flora between the genders [59]; and use of a-antagonists, which can increase the surgical complexity, as they can lead to intraoperative floppy iris syndrome [24].

Although small-incision phacoemulsification has remained the standard of care, surgeons continue to debate whether modifications in surgical technique have affected complication rates. Our pooled analysis of six studies confirmed the increased risks of acute POE associated with ECCE/ICCE compared with phacoemulsification surgery from both developed and developing countries. It was consistent with the reports of western Australia in 2011 [60] and southern India in 2009 [61]. While other studies have found on difference [62 ∼ 64] or the opposite conclusion [43] comparing postoperative complication rates with the transition from ECCE to phacoemulsification techniques. In a setting with phacoemulsification as the standard method, a selection bias for ECCE/ICCE in particularly difficult cases, e.g. instrumental surgical intervention for mature and hypermature cataracts is possible to lead to some complications concerning zonular fiber damaging, is introduced that may very well influence the results. The larger incision and the longer duration of the operation in ECCE than in phacoemulsification, together with the use of perioperative intracameral antibiotics in the phacoemulsification operation may explain this difference.

Is a clear corneal incision associated with greater odds of endophthalmitis compared with a scleral tunnel or limbal incision? Controversy exists regarding the problem. Theories to account for more frequent POE with sutureless clear corneal incisions are centered on the stability of the surgical wound because its integrity is believed to be a critical factor. A stable, self-sealing incision may be technically more difficult in the cornea than in the sclera. Many reports concluded that postoperative wound defects were a risk factor for the development of endophthalmitis [65], [66] and the corneal incision at least 2.0mm in length had substantially greater resistance to incision failure [45]. This suggests that the integrity of a self-sealing incision depends to some extent on length. This may be more difficult in a clear corneal incision. If the incision is too short, the cataract wound may be susceptible to a postoperative perturbation (such as rubbing of the eye) and wound abnormality. According to the innovations in phacoemulsification technology, the types of instruments available to better manage complex cases (pupil stretchers, capsular tension rings, dyes to stain the capsule), increased use of topical anesthesia, improvements in intraocular lenses, changes in preoperative or postoperative medication regimens, and better strategies to deal with intraoperative complications, two more recent studies showed the rates of adverse events, including endophthalmitis, decreased among patients undergoing small-incision phacoemulsification from 1994 to 2006 [22], [67]. The incision location, structure and length should be more thoroughly studied in large prospective trials in the future.

Three multicenter prospective randomized partially masked control study concurred that the most pertinent finding of the protective effect against infection produced by the prophylactic use of intracameral cefuroxime (1 mg in 0.1 ml solution) compared with topical disinfection alone [28], [31], [35]. A current prospective observational study reported the intracameral cefazolin (1 mg in 0.1 ml solution) significantly reduced the rate of postoperative endophthalmitis. The magnitude of the ORs shown by our meta-analysis were inconsistent across studies while the pooled estimates were statistically significant for both without intracameral cefuroxime (OR 5.48, 95% CI 3.79∼7.92) and without intracameral cefazolin (OR 10.76, 95% CI 6.45∼17.95) with no evidence of heterogeneity (I^2^ = 48.6%, *P* = 0.083; I^2^ = 0%, *P* = 0.858 respectively). Coagulase-negative staphylococcus (shown in Table 2) is the most commonly isolated organism and is followed by other gram-positive organisms (such as staphylococcus aureus, streptococcus species) and gram-negative bacteria. Cefuroxime or cefazolin is usually effective against the broad spectrum of bacteria causing acute onset postoperative endophthalmitis. Endophthalmitis caused by coagulase-negative staphylococci may have less inflammatory signs, often creating difficulty in distinguishing between an infective and a noninfective etiology. Many surgeons fear an increased incidence of toxic anterior segment syndrome (TASS) with injected antibiotics, such as the toxic effects of higher concentrations of cefuroxime and vancomycin on human corneal endothelial cells [70]. More research on the clinically used concentrations was recommended.

Our meta-analysis confirmed the increased risks of acute endophthalmitis associated with silicone lOLs. This seems to corroborate experimental studies [55], [68], [69] and also some clinical data, reporting an increased bacterial adhesion to silicone lenses compared with polymethylmethacrylate lOLs and hydrophobic acrylic IOLs, as the first-line implants in most operating practice due to the favoring of foldable IOLs to avoid induction of astigmatism [74∼76]. The future new lens materials or design may confer greater resistance of intraocular organisms to physiological and pharmacological antibacterial protective mechanisms. Evaluation on the uveal and capsular biocompatibility shape of IOL should also be considered to prohibit lens epithelial cell migration and postoperative inflammation.

Posterior capsular rupture caused intraoperative communication with the vitreous cavity, was found to be a significant risk factor for postoperative endophthalmitis, which was well proved by in vitro experiments [71] and animal models [72], [73]. Our pooled estimates revealed that PCR was associated with an increased risk of more than six-fold for acute endophthalmitis. This risk increased when we excluded the two studies that had a lower cut-off for prospective design. When other intraoperative complications were added, the pooled estimates OR was 5.28 (95% CI, 2.74∼10.18), suggesting that PCR may be the common intraoperative risk and do most contribution to the incidence of endophthalmitis.

There are several strengths in our systematic review. We performed a comprehensive search through six databases, had inclusion criteria for the prospective, cross-sectional and retrospective studies. The fully adjusted study-specific ORs were combined to estimate the pooled ORs with 95% CI using the random effects model for analyze the heterogeneity. The uniquely large sample size and inclusion of studies from different ethnic populations around the world could provide a more precise estimate of the perioperative risk factors for POE in the general population because they included known, presumably symptomatic, and unknown risks.

There are potential limitations to the present literature synthesis, some inherent to systematic reviews in general and some particular to our review. First, the studies included in this analysis may be subject to some methodological variation. Definitions of endophthalmitis may have varied; in addition, inherent difficulties in the diagnosis of this complication are apparent secondary to the uncommon manifestation of the “classic” form of postsurgical endophthalmitis. Miscoding of endophthalmitis itself could be a serious concern for data quality of any epidemiological analysis. Second, the overwhelming number of publications showing retrospective data, and the limited number of prospective and case-controlled studies with appropriate randomization methods, negatively affected the proportion of high-quality articles reviewed. Systematic reviews have an intrinsic limitation: the quality of the outcome depends on the quality of the inputs. Therefore, their findings must be interpreted with caution. Nonetheless, many studies included in this review were from Asian populations (e.g. Chinese, Malay Asians, Thailand, India) and thus, we believe our results can be generalizable to different populations in different countries around the world. Finally, the major setback of published studies and meta-analyses of published studies in general is publication bias. Publication bias may be an issue because studies that report statistically significant results are more likely to get published than studies that report nonsignificant results, and this could have distorted the findings of our meta-analyses. Therefore, potentially additional unpublished evidence regarding risk factors of acute endophthalmitis following cataract surgery during the past decade may be unavailable for analysis [4]. However, Egger regression asymmetry test and the Begg's test suggested no evidence of publication bias in our study.

Nonetheless, even with these limitations in mind, we believe that our analysis provides clear evidence to support the notion that the nine risk factors for acute endophthalmitis. This study provides additional information for primary care physicians, general ophthalmologists and other eye care professionals to counsel their patients on acute POE risk.

## Supporting Information

Checklist S1 **PRISMA Checklist.**
(DOC)
